# Functional outcomes by reconstruction technique following laparoscopic proximal gastrectomy for gastric cancer: double tract versus jejunal interposition

**DOI:** 10.1186/1477-7819-12-20

**Published:** 2014-01-27

**Authors:** Eiji Nomura, Sang-Woong Lee, Masaru Kawai, Masashi Yamazaki, Kazuhito Nabeshima, Kenji Nakamura, Kazuhisa Uchiyama

**Affiliations:** 1 Department of Gastroenterological and General Surgery, Tokai University Hachioji Hospital, 1838 Ishikawa-machi, Hachioji, Tokyo 192-0032, Japan; 2 Department of General and Gastroenterological Surgery, Osaka Medical College, 2-7 Daigaku-machi, Takatsuki, Osaka 569-8686, Japan; 3 Department of Gastroenterological and General Surgery, Tokai University School of Medicine, 143 Shimokasuya, Isehara, Kanagawa 259-1193, Japan

**Keywords:** Gastric cancer, Laparoscopic proximal gastrectomy, Double tract reconstruction, Jejunal interposition reconstruction, Quality of life

## Abstract

**Background:**

For early gastric cancer located in the upper third of the stomach, we have adopted laparoscopic 1/2-proximal gastrectomy (PG) with two types of reconstruction: double tract reconstruction (L-DT) and jejunal interposition reconstruction with crimping of the jejunum on the anal side of the jejunogastrostomy with a knifeless linear stapler (L-JIP).

**Methods:**

Functional outcomes were prospectively compared between these two types of reconstruction following laparoscopic PG. Resection and reconstruction were performed using L-DT (n = 10) and L-JIP (n = 10) alternately. Quality of life was evaluated through a questionnaire and endoscopic examination of the ten patients in each group, and functional evaluations were carried out in five patients of each group.

**Results:**

The postoperative/preoperative body weight ratio was significantly higher in the L-JIP group than in the L-DT group. While the incidence of reflux esophagitis was 10% in both groups, the endoscope could reach the remnant stomach in all patients. In the L-DT group, the plasma acetaminophen concentration at 15 minutes and the insulin level at 30 minutes were markedly increased after oral administration, while the increases in the blood sugar level at 30 and 60 minutes were more gradual than in the L-JIP group.

**Conclusions:**

While L-JIP may be thought of as the ideal method for function-preserving gastrectomy, L-DT may be suitable for gastric cancer patients with impaired glucose tolerance. These results raise the possibility of individualized selection of reconstruction for gastric cancer patients with various kinds of preoperative complications.

## Background

The incidence of early gastric cancer has increased in recent years [1]. Since patients are expected to survive for longer after surgery, there has been increasing demand for less invasive and safer operative procedures that are associated with improved postoperative quality of life (QOL) [2]. For early primary gastric cancer located in the upper third of the stomach, we perform proximal gastrectomy (PG). Various methods of open or laparoscopic resection with reconstruction have been devised over time [3-5]. Standard PG for early cancer, as defined by the Japanese gastric cancer treatment guidelines [6], requires resection of less than half of the stomach. The criteria for PG in our institute were: 1) a primary tumor located in the upper one-third of the stomach; 2) cancerous invasion not extending beyond the submucosal layer (T1); and 3) no macroscopic evidence of lymph node metastasis (N0) at the time of surgery [7,8]. Recently, laparoscopic gastrectomy and reconstruction have been adopted as a potentially less invasive surgical approach [9,10]. We have recently been performing laparoscopic PG for early gastric cancer, with reconstruction by the double tract (DT) method. However, when we performed open PG, the jejunal interposition method (JIP) was adopted and contributed to better quality of life for the patient, especially reduction of postoperative body weight loss compared to that after jejunal interposition following total gastrectomy and subtotal proximal gastrectomy [11]. Therefore, we devised a method to change to laparoscopic JIP (L-JIP) from laparoscopic DT (L-DT) by crimping the jejunum on the anal side of the jejunogastrostomy with a knifeless linear stapler.

In this study, functional outcomes were prospectively compared between L-DT and L-JIP reconstruction following laparoscopic 1/2-proximal gastrectomy for gastric cancer. Which reconstruction could maintain better QOL following proximal gastrectomy was also examined.

## Methods

This study evaluated a total of 20 patients who underwent laparoscopic PG for cancer between April 2010 and June 2012 at our institution. Resection and reconstruction were prospectively performed using L-DT and L-JIP alternately. This was accompanied by dissection of perigastric lymph nodes up to D1+ (dissection of lymph node stations 7, 8a, 9, and 11p in addition to the perigastric nodes) [12]. The hepatic and pyloric branches of the vagus nerve were routinely preserved, but preservation of the celiac branch was not considered. Clinicopathological findings of the gastric resections were recorded according to the Japanese classification of gastric carcinoma, 3rd English edition [13].

The primary outcome measure was postoperative digestive function measured by the postoperative/preoperative body weight ratio, postoperative/preoperative meal intake ratio, and the degree of postprandial abdominal symptoms. The postoperative/preoperative meal intake ratio was indicated approximately by the mean of the whole meal intake per day as compared to the preoperative intake. These data were acquired at one time point, 12 months postoperatively, through an in-house questionnaire (Table 1). In addition, the findings of patients who underwent endoscopy postoperatively at our outpatient clinic were analyzed to investigate the incidence of esophagitis. Endoscopic findings of esophagitis were categorized by the Los Angeles classification [14].

**Table 1:** Questionnaire survey about postoperative body weight, meal intake, and abdominal symptoms

1. Please describe your body weight at present	Kg
2. Please put a circle around the number below that fits your present postoperative whole amount of meal intake per day compared to your preoperative whole meal intake.	
1) 20%	
2) 40%	
3) 60%	
4) 80%	
5) 100%	
6) Other	%
3. Please put a circle around the number below that fits your description of abdominal symptoms often occurring especially after meals at present.	
1) Borborygmi	
2) Abdominal pain	
3) Diarrhea	
4) Nausea, or Vomiting	
5) Abdominal sensation of feeling full	
6) Abdominal discomfort	
7) Heart burn, or Reflux	
8) No symptoms	

In addition, functional evaluation was performed for patients who were undergoing regular follow-up at our hospital and agreed to be involved in the study. The course of intestinal absorption and gastric non-absorbable stasis was investigated with acetaminophen (AAP) in five L-DT group patients and five L-JIP group patients, excluding patients with impaired glucose tolerance. AAP is not absorbed in the stomach but is absorbed in the duodenum or jejunum, through which it enters the blood stream [15]. Patients swallowed an alimentary liquid (200 mL of Ensure liquid mixed®, Meiji, Tokyo, Japan) containing 1.5 g of AAP, and the concentration of AAP in the blood was measured every 15 minutes for 60 minutes [5,11]. At the same time, the blood concentrations of sugar, insulin, and gastrin were measured.

This study protocol was approved by the Human Ethics Review Committee of Osaka Medical College. Written, informed consent was obtained from each enrolled patient before study entry in accordance with the Declaration of Helsinki.

### Surgical procedures

For proximal 1/2-gastrectomy, the resection line was, in principle, at 10 cm along the lesser curvature and 15 cm along the greater curvature as measured from the pyloric ring. The tumor was confirmed as being located in the upper third of the stomach preoperatively and intraoperatively. This was often ascertained through preoperative upper gastrointestinal series or endoscopic submucosal tattooing with 0.1 mL of India ink. Two types of reconstruction following PG were performed alternately: laparoscopic proximal 1/2-gastrectomy followed by double tract reconstruction with a 6-cm jejunogastrostomy (L-DT), and laparoscopic proximal 1/2-gastrectomy followed by jejunal interposition reconstruction by crimping the jejunum on the anal side of the jejunogastrostomy in L-DT with a knifeless linear stapler (L-JIP).

L-DT was performed by interposing a 15-cm segment of jejunum between the esophagus and residual stomach. In brief, the anvil head of the circular stapler (PCEEA™ (Covidien, Mansfield, MA, USA)) was inserted into the esophageal stump. The jejunum was divided 20 cm distal to the ligament of Treitz. A side-to-side jejunojejunostomy was created by an anastomosis between the divided oral jejunum and 30 cm of anal jejunum from the oral jejunal stump. An entry hole for the circular stapler was made halfway (15 cm) along the anal jejunal stump, and the circular stapler was used to achieve esophagojejunostomy intracorporeally. After connecting the anvil head of the stapler and the circular stapler, an end-to-side esophagojejunostomy was fashioned. In order to clearly observe the anastomotic site without being disturbed by the circular stapler inserted through an umbilical port wound, it was thought better to insert the circular stapler through the entry hole that made into the jejunogastrostomy subsequently.

**Figure 1:**
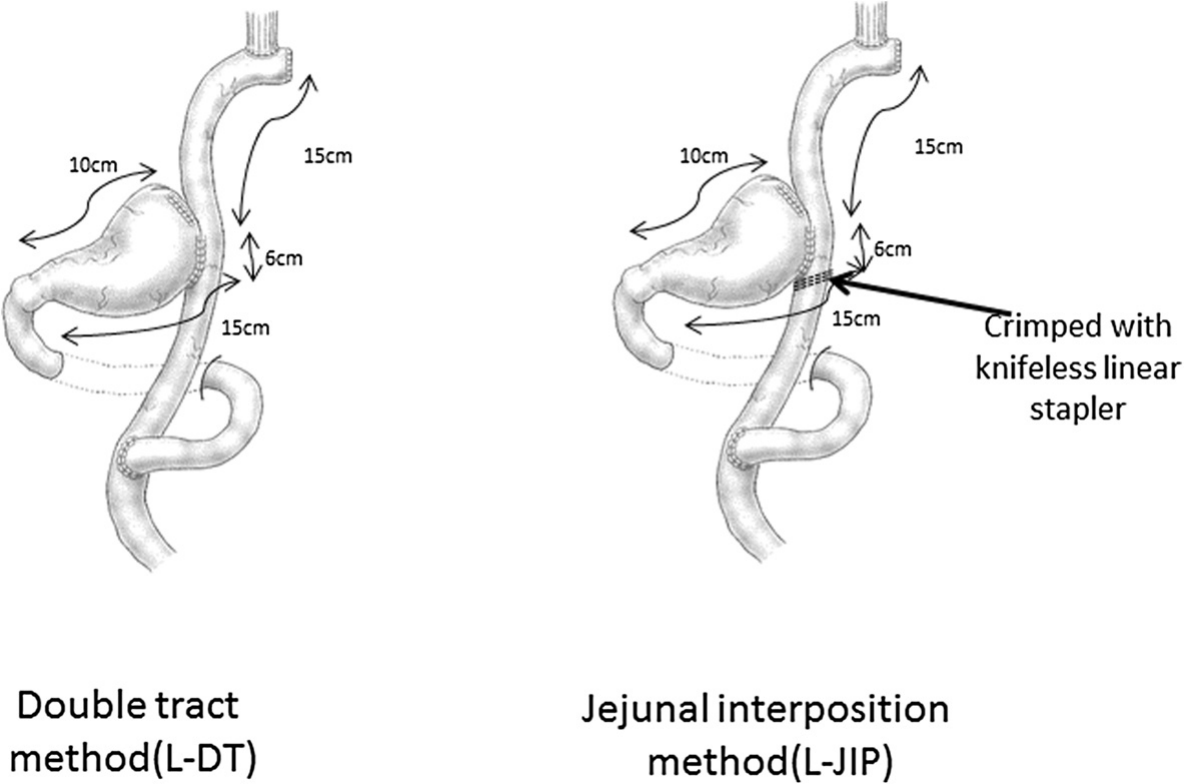
**Schematic illustrations of the surgical procedures.** L-DT: laparoscopic double tract reconstruction following proximal gastrectomy. L-JIP: laparoscopic jejunal interposition reconstruction following proximal gastrectomy.

After removing the circular stapler, the anastomosis between the entry hole and the oral edge of the remnant stomach was made by hand sewing through an umbilical wound. The length of the jejunogastrostomy was 6 cm. For L-JIP, the jejunum on the anal side of the jejunogastrostomy was then crimped with a knifeless linear stapler. These procedures are illustrated in Figure 1.

Statistical analysis was performed using Student’s *t*-test and the *χ*^2^ test. A *P*-value of less than 0.05 was considered significant.

## Results

Of the 20 patients who underwent laparoscopic PG, ten patients underwent L-DT, and ten patients underwent L-JIP. All patients completed the digestive function questionnaires. Patient demographics, stratified according to the surgical procedure, are presented in Table 2; there were no significant differences between the two groups. Follow-up revealed that there was no evidence of recurrence at one year after surgery in any patient.

**Table 2:** Characteristics of patients by type of reconstruction

**Reconstruction**	**Sex (male: female)**	**Age (years)**	**Depth of invasion (m/sm/mp)**	**Lymph node metastasis (n0/n1)**	**Stage (IA/IB/II)**
L-DT (n = 10)	8:2	65.8 ± 10.3	3/6/1	n0:9 n1:1	9/0/1
L-JIP (n = 10)	7:3	68.5 ± 6.2	2/6/2	n0:9 n1:1	8/1/1

Legend: m, mucosa; sm, submucosa; mp, muscularis propria.

### Functional outcomes at 12 months

**Figure 2:**
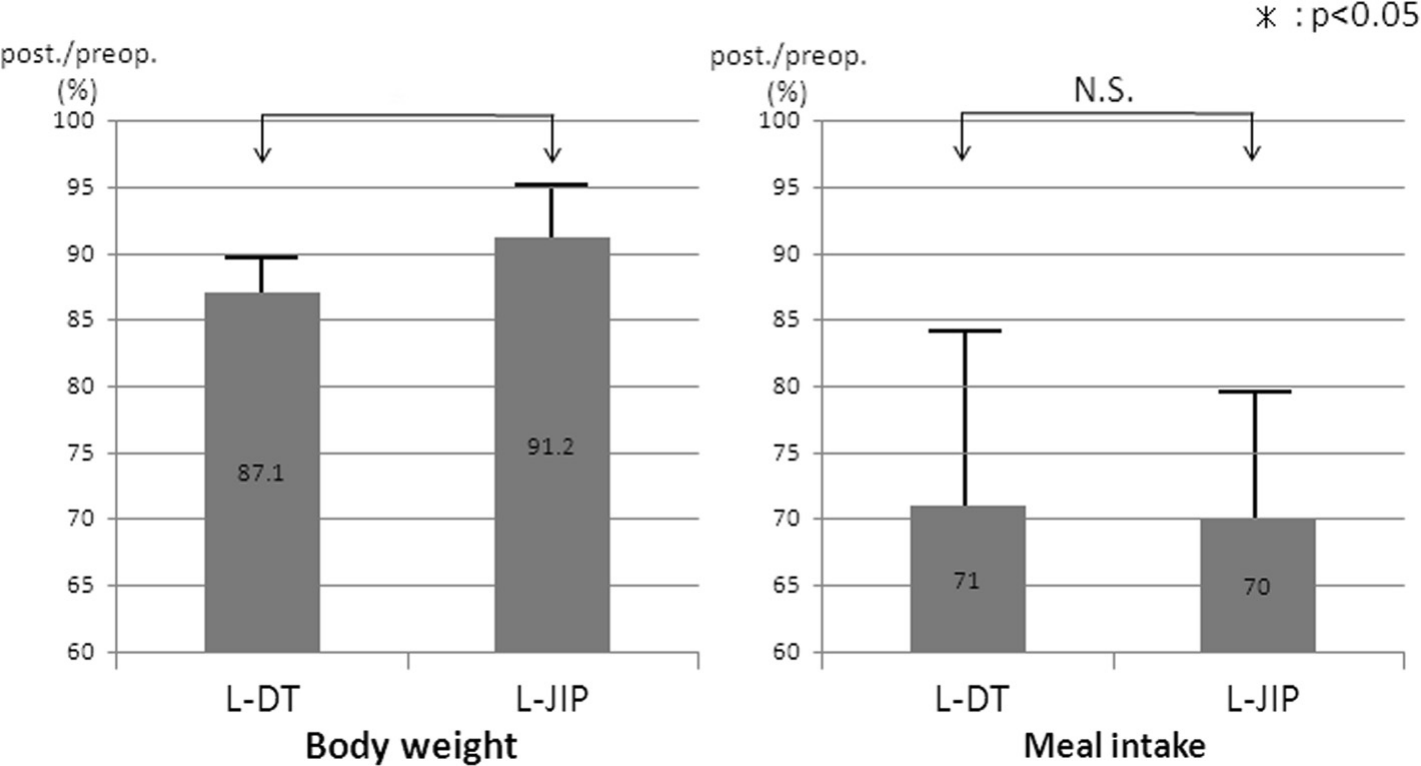
**Postoperative/preoperative body weight and meal intake ratios.** Postoperative/preoperative body weight ratios are significantly higher in the laparoscopic jejunal interposition (L-JIP) group than in the laparoscopic double tract (L-DT) group (*P < 0.05).

While comparison of the postoperative/preoperative meal intake ratio (Figure 2) revealed no significant difference between the two groups, the postoperative/preoperative body weight ratio was significantly higher in the L-JIP group than in the L-DT group (Figure 2). With respect to postprandial symptoms, a heavy abdominal feeling was frequent in both groups: 20% (2/10) in L-DT, 30% (3/10) in L-JIP. Borborygmi (20%, 2/10) were frequent, and a full abdominal sensation was reported in one patient in the L-JIP group. Heartburn, nausea, abdominal pain, and borborygmi were observed in one patient each in the L-DT group. However, there were no significant differences between the two groups.

The incidence of reflux esophagitis on endoscopic examination in both groups was 10% (1/10). Stenosis of the esophagojejunostomy was observed in one patient in the L-DT group and two patients in the L-JIP group, but these stenoses were improved by a single balloon dilatation. The endoscope could reach the remnant distal stomach in all patients.

**Figure 3:**
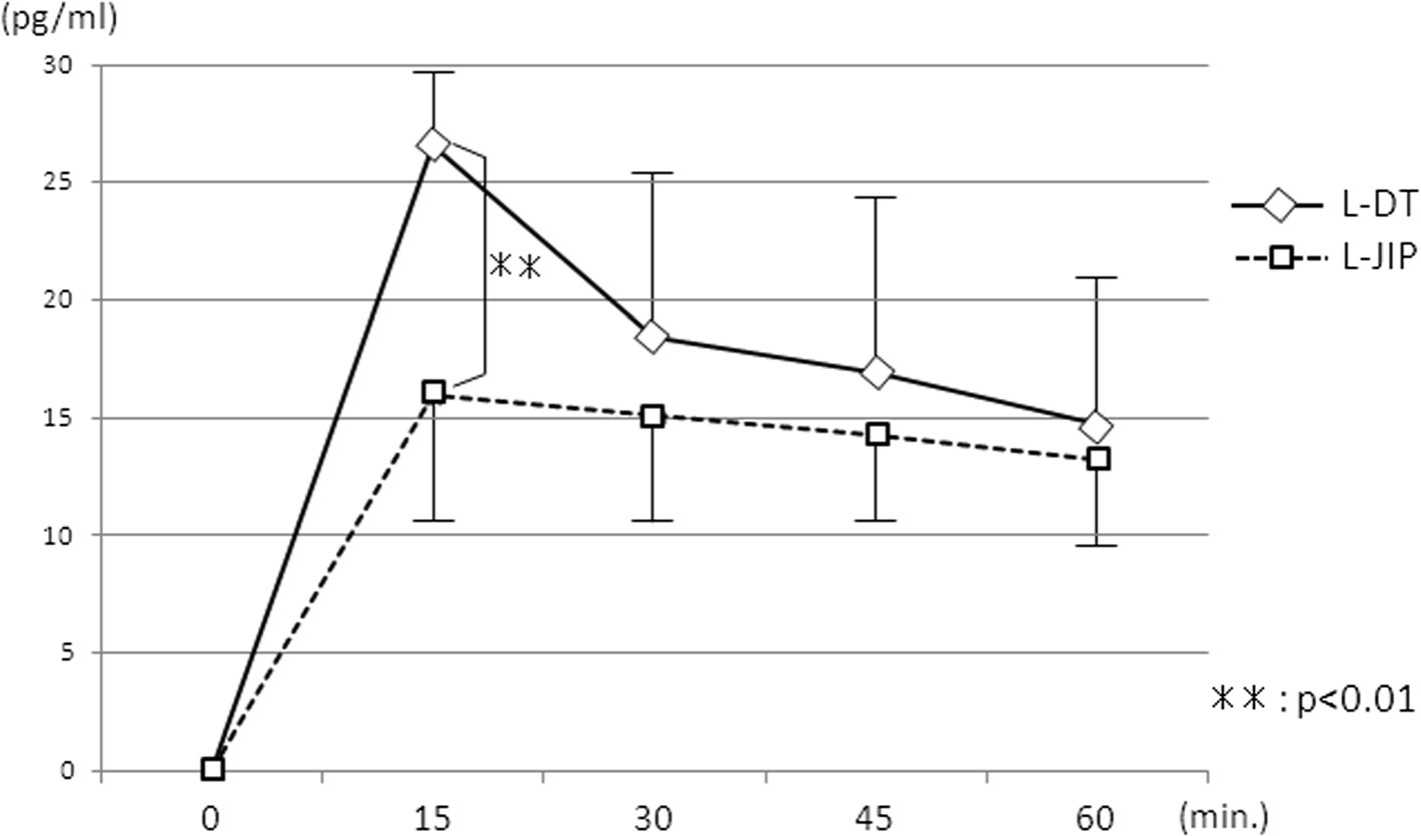
**Changes in plasma acetaminophen concentrations.** **P < 0.01.

**Figure 4:**
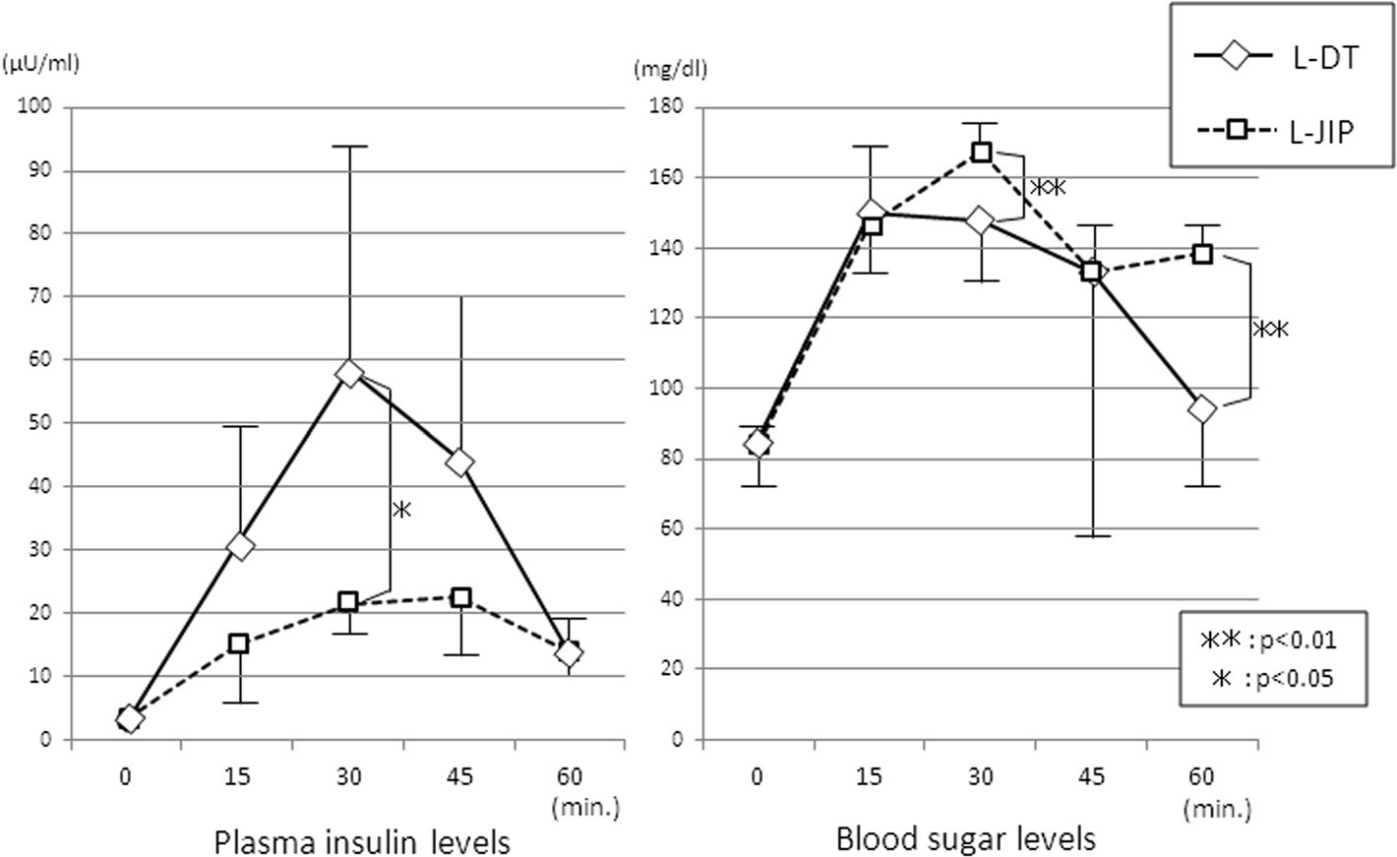
**Changes in postprandial insulin and glucose levels.** **P < 0.01, *P < 0.05.

**Figure 5:**
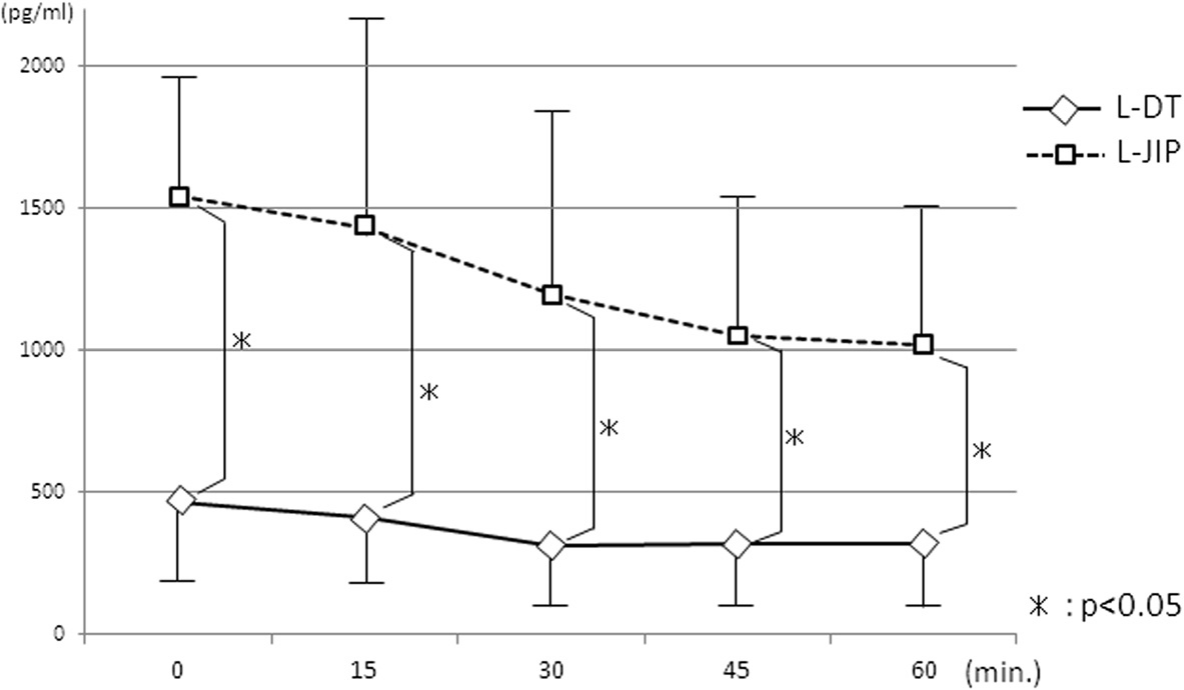
**Changes in plasma gastrin levels.** *P < 0.05.

The plasma AAP concentration at 15 minutes after oral administration was significantly higher in the L-DT group than in the L-JIP group (Figure 3). The insulin level at 30 minutes was markedly increased in the L-DT group, while the increases in the blood sugar level at 30 and 60 minutes were more gradual in the L-DT group than in the L-JIP group (Figure 4). Furthermore, the plasma gastrin level was much higher in the L-JIP group than in the L-DT group (Figure 5).

## Discussion

Most patients with advanced gastric cancer in the upper one-third of the stomach have poor prognoses and undergo total gastrectomies or combined resections with splenectomy [16]. Because the rate of lymph node metastasis for early gastric cancer in the upper third of the stomach is low, a more conservative surgical approach in accordance with the early stage of the cancer should be selected, similar to treatment for gastric cancer in the lower or middle third of the stomach [7].

PG for gastric cancer is thought to be one of the limited approaches to surgery that preserve the distal stomach, pyloric ring, and vagus nerve. Moreover, if PG is considered limited surgery, the laparoscopic approach could be adopted as a feasible and potentially less invasive surgical approach. For early gastric cancer located in the upper third of the stomach, we have adopted laparoscopic PG and double tract reconstruction, since these procedures are relatively simple [7]. However, when we performed open PG, the jejunal interposition method was adopted and contributed to better QOL for the patients [11]. Therefore, we devised a method to change to L-JIP from L-DT by crimping the jejunum on the anal side of the jejunogastrostomy with a knifeless linear stapler. Physiological meal passage through the duodenum might contribute to reduction of postoperative body weight loss. However, there is little evidence about the nutritional advantages due to meal passage through the duodenum [17]. The reduction of postoperative body weight loss in this study was thought to be one of the most important factors related to maintaining good QOL, and it was likely the result of better digestive and absorptive functions.

Gastric cancer treatment guidelines call for the use of PG only when, for T1, N0 gastric tumors in the upper one-third of the stomach [6], more than one-half of the distal stomach can be preserved. Namely, the extent of gastrectomy and the degree of lymph node dissection have almost been determined, and only the approaches and reconstruction methods remain to be determined. Because the indication for PG is confined to early gastric cancer, the laparoscopic approach is thought to be feasible and reconstruction to maintain better QOL is desired. In our previous analysis of open surgery for early gastric cancer, reduction of the extent of gastrectomy and preservation of the vagal branches and of the pyloric ring were associated with better QOL [11]. In particular, we think that reduction of the extent of gastrectomy is the most important factor [7]. Thus, the jejunal interposition reconstruction is thought to be the ideal method to fulfill all three criteria.

Furthermore, the reconstruction following PG should prevent reflux esophagitis and allow observation of the remnant stomach through endoscopy; for this, five criteria must be satisfied. Although the occurrence rate of reflux esophagitis was 10%, the result that the remnant stomach could be observed in all cases indicates that a 15-cm interposed jejunal segment was appropriate. The incidence rate of stenosis of the esophagojejunostomy (EJ) was 10% in the L-DT group and 20% in the L-JIP group. However, the number of patients examined in this study was too small to evaluate abdominal symptoms and the occurrence rate of anastomotic stenosis and reflux esophagitis in detail; therefore, further clinical trials comparing L-DT and L-JIP will be needed.

Fukagawa *et al*. reported that the incidence rate of EJ stenosis following open PG was 7.0% [18]. Multivariate analysis identified female sex, PG, and the use of a 21-mm stapler as independent risk factors for anastomotic stenosis. Because almost all cases of PG were performed by jejunal interposition in their institute, they suggested that the reason for the high incidence of EJ stenosis in PG cases needs to be investigated in a future study. In the present study, a higher rate of EJ stenosis was observed in L-JIP reconstruction following laparoscopic PG, and further investigation is needed, although endoscopic treatment yielded favorable outcomes.

Because it is necessary for function-preserving gastrectomy that the postprandial hormonal secretion resembles its preoperative status, we have used this examination as a method to evaluate postoperative function [11]. It was found that the increases in acetaminophen and insulin levels were significantly higher in the L-DT group than in the L-JIP group. On the other hand, the changes in blood sugar levels were less marked in the L-DT group than in the L-JIP group. These results are similar to those seen with bariatric surgery for morbid obesity, which causes insulin secretion or sensitivity to improve. It is thought that hormones such as incretins secreted from the small intestine accelerate insulin secretion and suppress the changes in blood sugar [19,20]. Incretins are a group of gastrointestinal hormones that increase the amount of insulin released from the beta cells of the islets of Langerhans after eating, even before blood sugar levels become elevated. They also slow the rate of absorption of nutrients into the blood stream by reducing gastric emptying and may directly reduce meal intake. As expected, they also inhibit glucagon release from the alpha cells of the islets of Langerhans. The two main candidate molecules that fulfill criteria for an incretin are glucagon-like peptide-1 (GLP-1) and gastric inhibitory peptide (GIP). Both GLP-1 and GIP are rapidly inactivated by the enzyme dipeptidyl peptidase-4 [20].

The form and volume of a loading meal might affect hormonal secretion, so it is very difficult to determine which reconstruction is better in terms of hormonal secretion. In the present study, the L-DT method might be considered suitable for gastric cancer patients with impaired glucose tolerance. These results raise the possibility of individualized selection of reconstruction for gastric cancer patients with various kinds of preoperative complications.

There remain questions about the gut hormone gastrin. In PG, especially L-JIP, hypergastrinemia was characteristically found. This hypergastrinemia in PG was likely caused by a negative feedback mechanism in which the gastrin secretion area was preserved, and the acid secretion area was resected. It has yet to be determined whether hypergastrinemia has a good effect. Gastrin also acts as a potent cell-growth factor that has been implicated in a variety of normal and abnormal biological processes, including maintenance of the gastric mucosa, proliferation of enterochromaffin-like cells, and neoplastic transformation [21]. Further investigation of the effects of gastrin in L-JIP with severe hypergastrinemia is needed. Although PG has been investigated for a long time, there have been many questions and problems to solve. PG requires much work, but more investigations are needed to maintain better QOL following gastrectomy.

## Conclusions

While L-JIP may be thought to be the ideal method for function-preserving gastrectomy, given the results of the present study, L-DT may be suitable for gastric cancer patients with impaired glucose tolerance. These results raise the possibility of individualized selection of reconstruction for gastric cancer patients with various kinds of preoperative complications. Further randomized clinical trials comparing L-DT and L-JIP will be needed to verify various functions in detail, including investigations of hormones such as incretins.

## Abbreviations

QOL: quality of life; PG: proximal gastrectomy; T1: cancerous invasion not extending beyond the submucosal layer; DT: double tract; JIP: jujunal interposition; L-DT: laparoscopic double tract; L-JIP: laparoscopic jejunal interposition; AAP: actaminophen; EJ: esophagojejunostomy; GLP-1: glucagon-like peptide-1; GIP: gastric inhibitory peptide.

## Competing interests

The authors declared that they have no competing interest.

## Authors’ contributions

EN designed and conducted the study, analyzed the data, and helped to write the manuscript. SL and MK helped to design the study, conducted surgical operations, and helped to write the manuscript. MY, KN, and KN helped to design the study and helped to write the manuscript. KU is the principal investigator, designed the study, and assisted in writing, revising, and editing the manuscript. All authors approved the final manuscript.
